# Overcoming drug resistance in glioblastoma: new options in sight?

**DOI:** 10.20517/cdr.2021.03

**Published:** 2021-06-19

**Authors:** Joanna Kopecka, Chiara Riganti

**Affiliations:** Department of Oncology, University of Torino, Torino 10126, Italy.

Glioblastoma (GB) represents 48% of all primary central nervous system (CNS) tumors. Despite the multimodal therapy, based on surgery, radiotherapy, and chemotherapy, and the improvements in each approach, GB remains characterized by a poor prognosis, with a mean overall survival of less than 6% at 5 years post-diagnosis^[1]^. Currently, the first-line systemic treatment is represented by temozolomide (TMZ), but most patients relapse after six months. Re-challenge with TMZ or administration of lomustine, carmustine, vincristine, procarbazine, or anti-angiogenic drugs such as bevacizumab are rescue therapies, but they have low success^[1]^.

Resistance in GB is a multifactorial process. The methylation status of O^6^-methylguanine-DNA methyltransferase (MGMT) has been the first factor implicated in the resistance to TMZ, but this feature alone - although useful in discriminating the treatment options^[1]^ - does not predict the efficacy of TMZ at clinical level^[2]^. The presence of cancer stem cells (SCs) is another cause of chemoresistance^[3,4]^, since this component is particularly hard to be eradicated pharmacologically^[5]^. Several amplifications and activating mutations in common oncogenes, such as epithelial growth factor receptor (EGFR), platelet-derived growth factor receptor A (PDGFRA), and isocitrate dehydrogenase 1 (IDH1), together with deletions or inactivating mutations in onco-suppressor genes, such as phosphatase and tensin homolog (PTEN), TP53, and cyclin-dependent kinase inhibitor 2A (CDKN2A), have been reported as negative prognostic factors^[6]^. However, the identification of a molecular signature indicative of drug sensitivity or resistance is still a matter of debate^[7]^. Building a combined classification of phenotypic, genetic, molecular, and epigenetic profiles of patients, exploiting the recent advancement in next generation sequencing and artificial intelligence, is an attractive option to achieve the goal of a personalized treatment in GB. However, this approach is currently limited by low accessibility to patient data, data protection issues, and technical difficulties encountered in the training and validation of machine learning-based algorithms^[8]^. The presence of a high inter- and intra-GB heterogeneity and the intrinsic plasticity of GB cells to acquire new mutations are additional factors making difficult the precise prediction of drug resistance^[3,9]^ and the consequent precision medicine for GB patients. Finally, the GB immune environment has recently been evaluated as a factor linked to tumor aggressiveness. Indeed, the prevalence of immune-suppressive cells, such as M2-polarized tumor-associated macrophages, myeloid-derived suppressor cells, T-regulatory cells, and PD1-positive CD8^+^ T-lymphocytes, favors GB relapse and drug resistance^[9]^. At the same time, the high mutational burden^[10]^ and the peculiarities of GB immune environment may pave the way to new immune-therapy-based approaches, such as immune checkpoint inhibitors, dendritic cell-based vaccination, or CAR T-cells^[9,10]^. However, while in the GB bulk the brain - blood barrier (BBB) is disrupted, it is often intact in the brain-adjacent-to-tumor (BAT) area, where the single GB cells present are the main source of recurrence^[11]^. Such BBB integrity makes unpredictable the penetration of chemotherapy and the efficacy of immune therapy approaches.

In consideration of the pleiotropic mechanisms of resistance, the limitations in stratifying patients, and the few treatment options available, new strategies overcoming drug resistance in GB are urgently needed. In this Special Issue, we provide an overview on the latest developments in this field.

Dormancy or quiescence has been associated with chemoresistance in several tumors. This aspect was also investigated in GB, where the presence of sensitive, primary resistant, and secondary resistant cells is common, as a consequence of the tumor heterogeneity^[3,9]^. After the first cycles of chemotherapy and the elimination of the sensitive cells, it has been demonstrated that small populations of “persister” cells, i.e., cells tolerant to the treatment, emerge in the residual tumor. These populations are phenotypically distinct from primary resistant cells, but together with the latter are gradually expanded by chemotherapy. Cells with secondary (or acquired) resistance may emerge from persister clones^[4]^. The coexistence of primary and secondary resistant cells, as well as of newly generated persister cells, determines a plethora of different drug resistance mechanisms, making GB difficult to be eradicated by a single agent such as TMZ. Persister cells are slowly cycling cells and show many phenotypic markers of GB SC, such as CD133 and Oct4 positivity, together with an increased activity of DNA repair machinery^[4]^. Therein, they easily resist TMZ-induced damages. In other solid tumors, dormancy is maintained by the aberrant activation of tyrosine kinase receptor pathways and epigenetic factors. Most importantly, the persister phenotype is reversible if cells are treated with tyrosine kinase inhibitors or chromatin remodeling agents, such as histone demethylase inhibitors. Since an altered activity of histone demethylase^[12]^ or histone deacetylase^[13]^ has been associated with TMZ resistance, the use of epigenetic drugs could be a new strategy in reversing the dormancy of GB cells and restoring the sensitivity to TMZ. Interestingly, persister cells show a higher dependence on glycolysis and fatty acid β-oxidation (FAO) than non-transformed neural cells or chemosensitive GB cells. This metabolic feature may be exploited by using glycolysis inhibitors, such as 2-deoxyglucose, or FAO inhibitors, such as etoxomir, as potential weapons to eradicate persister clones^[4]^. By studying the multiple features of persister cells, the Achille’s heels of these heterogeneously resistant clones can be unveiled, leading to test new combinatorial treatments that may re-transform dormant and resistant cells into sensitive ones.

The improvements in molecular profiling of GB patients are of paramount importance to identify new biomarkers of resistance to TMZ and new druggable targets. For instance, transforming growth factor β (TGF-β)/Smad pathway activates mitogen activated kinases (MAPKs) in GB, inducing resistance to chemotherapy. The small molecule OKN-007, an inhibitor of the TGF-β/Smad pathway, reverses TMZ resistance. On the one hand, OKN-007 limits tumor growth by preventing the remodeling of extracellular matrix. It synergizes with TMZ, reducing tumor growth and increasing patients’ overall survival^[6]^. Phosphatidylinositol-3 kinase (PI3K)/Akt axis is a second pathway often aberrantly activated in GB, determining increased cell survival and cell cycle progression. Sensitive and resistant patients to TMZ show a different activation of PI3K signaling, indicating that this parameter should be included among the factors predictive of response to TMZ. Interestingly, the highly specific PI3K inhibitor GDC-0941 chemosensitizes GB cells to TMZ^[6]^, supporting the hypothesis that PI3K activity is a driving factor in determining chemoresistance. Inhibitors of Akt/mammalian target of rapamycin (mTOR) such as sirolimus and everolimus have also been useful in reversing the resistance to EGFR inhibitors that - after an initial enthusiastic use - have revealed a high degree of resistance in GB^[6]^. Nuclear factor-kB (NF-κB), hepatocyte growth factor receptor (HGFR/c-Met), and Notch are other biomarkers often associated with poor response to TMZ and poor prognosis. These findings enlarge the number of small molecules that can be employed in reversing TMZ resistance, such as the NF-kB inhibitor parthenolide, c-Met inhibitors, and γ- or α-secretases inhibitors such as DAPT/GSI-IX, MRK-003, GSI-18, GW280164X, and INCB3619. Such small molecules may be evaluated as novel targeted therapies against resistant GB^[6]^.

One of the latest discoveries implicated in GB drug resistance is the mitochondrial melatonin synthesis catalyzed by aralkylamine N-acetyltransferase that transforms serotonin into N-acetyl-serotonin (NAS) and by NAS methyltransferase that produces melatonin. In GB cells, the reverse process, i.e., the synthesis of NAS from melatonin, may also occur, thanks to the activity of CYP1B1, a member of the cytochrome p450 family. Alterations in NAS/melatonin ratio determine tumor aggressiveness and resistance. For instance, an increased level of NAS activates the pro-proliferative pathways dependent on tyrosine receptor kinase B (TrkB), as well as the secretion of extracellular vesicles (EVs) rich in TrkB. This horizontal transfer contributes to induce a more aggressive phenotype in neighboring cells and to expand GB SC components^[14]^, explaining the well-known intra-tumor heterogeneity observed in GB^[9]^. Interestingly, GB cells also release kynurenine, a side product of the melatoninergic synthetic pathway. Kynurenine is an immune-suppressive mediator produced by several chemoresistant solid tumors^[15]^ and an activator of CYP1B1 in the microglia and macrophages infiltrating GB. Exploiting this crosstalk with the surrounding microenvironment, GB cells stimulate the production of NAS by immune-infiltrating cells, thereby contributing to maintain high the amount of GB SCs^[14]^. Moreover, melatonin inhibits the sphingosine 1-phosphate lyase (S1PL), maintaining a high ratio between the pro-survival lipid S1P and the pro-apoptotic lipid ceramide^[14]^. Increased cell survival and chemoresistance are associated with this phenotype.

Not only the immune-infiltrating cells but also the circulating ones are important in predicting the response to pharmacological treatments. In this Special Issue, the peripheral blood immune profile of patients treated with bevacizumab, alone or in combination with TMZ, was studied in subjects at high risk of progression. At the moment, there are no known factors for predicting the sensitivity to bevacizumab. In these high-risk patients, it was found that a high number of lymphocytes correlates with a better survival after bevacizumab treatment, while the simultaneous presence of high platelet-to-lymphocyte ratio (PLR) and high neutrophil-to-lymphocyte ratio (NLR) was associated with lower efficacy of bevacizumab and bad prognosis^[16]^. Although no data about the intratumor immune-infiltrate have been reported in this study, the results suggest a strong linkage between systemic inflammation and intratumor angiogenesis, opening the perspective of new combinatorial treatments - based on anti-inflammatory and anti-angiogenic drugs - as rescue therapies in patients relapsed after TMZ treatment.

Besides immune-infiltrating cells, another crucial component of the GB microenvironment are EVs. Resistant GB cells abundantly release EVs rich in mRNAs, miRNAs, and lncRNAs that increase drug efflux and drug inactivation. Once EVs released from resistant cells are transferred to sensitive cells, the latter acquire a resistant phenotype. Whole proteins, such as MGMT and drug efflux transporters such as P-glycoprotein (Pgp), both mediating resistance to TMZ, are also transferred via EVs and contribute to the “horizontal propagation” of resistance within the tumor mass^[3]^. EVs released from GB and enriched in these resistance factors are also detected in the serum of patients^[3]^. Their analysis can be useful in discriminating TMZ-responder versus non-responder patients and for identifying novel therapeutic approaches.

At present, notwithstanding the plethora of new treatment options experimented, none of them has been resolutive in overcoming resistance to TMZ, which is still the first pharmacological approach. Recently, an effective and minimally invasive tool against GB and many other solid tumors has been represented by photodynamic therapy (PTD), based on the irradiation of the tumor area, containing a photosensitizer agent, with visible light, able to trigger the release of reactive oxidative species (ROS) that kill tumor cells. Although in some cases nitric oxide (NO)-releasing photosensitizers have been successfully employed as anti-tumor^[17]^ and chemosensitizer^[18]^ agents, in GB cells, NO induces resistance to the oxidative damages elicited by PDT. Indeed, the production of NO by endogenous inducible NO synthase (iNOS) reduces the cell killing caused by PDT in GB cells pre-treated with protoporphirin IX as photosensitizer^[19]^. PDT itself induces iNOS that selects hyper-resistant and hyper-aggressive GB cells. This observation is one of the first cases of resistance to PDT reported in the literature. It cannot be excluded that a similar mechanism also occurs in other solid tumors. Conversely, disrupting such vicious cycle, by using competitive inhibitors of iNOS or inhibitors of iNOS transcription, may result in new therapeutic approaches that re-sensitize GB cells to PDT, turning hyper-resistant clones into hyper-sensitive ones^[19]^.

Overall, the mechanism of drug resistance in GB are multiple and interconnected, including both tumor-intrinsic factors and tumor microenvironment-dependent factors. A better multilayered profiling of patients, including detailed pathological, genomic, transcriptomic, and epigenetic data, should be developed to understand the specific resistance pathways of each patient. After this step, personalized medicine may be built on a rational basis. Such ideal therapy will likely be based on combinatorial treatments, given the multiple mechanisms of resistance and the high intra-tumor heterogeneity of GB. This approach requires the accumulation and analysis of big data, derived from thousands of patients, but it will be successful in the long term. It also represents a tremendous incentive to continue the research on drug resistance mechanisms, in order to implement chemosensitizing strategies for GB.

## Declarations

### Copyright

© The Author(s) 2021.
